# Use of communities of practice in business and health care sectors: A systematic review

**DOI:** 10.1186/1748-5908-4-27

**Published:** 2009-05-17

**Authors:** Linda C Li, Jeremy M Grimshaw, Camilla Nielsen, Maria Judd, Peter C Coyte, Ian D Graham

**Affiliations:** 1 Department of Physical Therapy, University of British Columbia; Arthritis Research Centre of Canada, Vancouver, Canada; 2 Ottawa Health Research Institute, Clinical Epidemiology Program, Centre for Best Practice, Institute of Population Health, University of Ottawa, Ottawa, Canada; 3 Centre for Health Technology Assessment, National Board of Health, Copenhagen, Denmark; 4 Canadian Health Services Research Foundation, Ottawa, Canada; 5 Department of Health Policy, Management and Evaluation Faculty of Medicine, University of Toronto, Toronto, Canada; 6 Canadian Institutes of Health Research, School of Nursing, University of Ottawa, Ottawa, Canada

## Abstract

**Background:**

Since being identified as a concept for understanding knowledge sharing, management, and creation, communities of practice (CoPs) have become increasingly popular within the health sector. The CoP concept has been used in the business sector for over 20 years, but the use of CoPs in the health sector has been limited in comparison.

**Objectives:**

First, we examined how CoPs were defined and used in these two sectors. Second, we evaluated the evidence of effectiveness on the health sector CoPs for improving the uptake of best practices and mentoring new practitioners.

**Methods:**

We conducted a search of electronic databases in the business, health, and education sectors, and a hand search of key journals for primary studies on CoP groups. Our research synthesis for the first objective focused on three areas: the authors' interpretations of the CoP concept, the key characteristics of CoP groups, and the common elements of CoP groups. To examine the evidence on the effectiveness of CoPs in the health sector, we identified articles that evaluated CoPs for improving health professional performance, health care organizational performance, professional mentoring, and/or patient outcome; and used experimental, quasi-experimental, or observational designs.

**Results:**

The structure of CoP groups varied greatly, ranging from voluntary informal networks to work-supported formal education sessions, and from apprentice training to multidisciplinary, multi-site project teams. Four characteristics were identified from CoP groups: social interaction among members, knowledge sharing, knowledge creation, and identity building; however, these were not consistently present in all CoPs. There was also a lack of clarity in the responsibilities of CoP facilitators and how power dynamics should be handled within a CoP group. We did not find any paper in the health sector that met the eligibility criteria for the quantitative analysis, and so the effectiveness of CoP in this sector remained unclear.

**Conclusion:**

There is no dominant trend in how the CoP concept is operationalized in the business and health sectors; hence, it is challenging to define the parameters of CoP groups. This may be one of the reasons for the lack of studies on the effectiveness of CoPs in the health sector. In order to improve the usefulness of the CoP concept in the development of groups and teams, further research will be needed to clarify the extent to which the four characteristics of CoPs are present in the mature and emergent groups, the expectations of facilitators and other participants, and the power relationship within CoPs.

## Background

One of the challenges to integrating research evidence into practice is that it involves a complex process of acquiring, converting, and applying a mix of explicit and tacit knowledge in clinical activities. Since being identified as a concept for understanding how people learn in a social environment [1-3], the community of practice (CoP) has been used by an increasing number of groups and teams in the health sector to help practitioners make sense of the concrete information (e.g. practice guidelines) in the context where it is used.

The concept of the CoP was originally developed by Lave and Wenger, who suggested that learning took place in social relationships rather than through the simple acquisition of knowledge [1]. To illustrate the concept, they used the example of how midwives, meat cutters, and tailors learned new knowledge relevant to their trades. Many of the exchanges of practical information and problem-solving happened during informal gatherings where tradesmen exchanged stories about their experience. Novices could also consult with experts in a non-threatening environment. Through this process, gaps in the practice were identified and solutions were proposed. Individuals might apply the solution in their own practice, and the outcomes were fed back to their colleagues for further refinement of the solution. Eventually these informal communications became the means for sharing information for improving practice and generating new knowledge and skills [1].

Lave and Wenger's observations have formed the basis of the 'situated learning theory,' which describes the learning that takes place in a setting functionally identical to that where the knowledge will be applied [1,4,5], thus contradicting the traditional learning activities that tend to isolate knowledge from practice. Later, Wenger proposed three interrelated dimensions to explain CoP: mutual engagement (the interaction between individuals that leads to the creation of shared meaning), joint enterprise (the process in which people are engaged and work together towards a common goal), and a shared repertoire (the common resources and jargon that members use to negotiate meaning within the group) [2].

In their latest publication, Wenger *et al*. refined the description of CoPs as 'groups of people who share a concern, a set of problems, or a passion about a topic, and who deepen their knowledge and expertise in this area by interacting on an ongoing basis' [6]. They identified three essential characteristics of CoPs: domain, community, and practice. The 'domain' creates common ground (i.e. the minimal competence that differentiates members from non-members), and outlines the boundaries that enable members to decide what is worth sharing and how to present their ideas. The 'community' creates the social structure that facilitates learning through interactions and relationships with others. The 'practice' is the specific knowledge that the community shares, develops, and maintains. Wenger *et al*. purport that a well-developed CoP group (i.e. when the three elements work well together) provides an environment that facilitates learning and knowledge development [3], but the literature is less clear on how to foster the three elements, especially at the early stage.

To improve the understanding about the use of the CoP concept, we conducted a research synthesis project to explore how the concept was operationalised in the business and health sectors. The objective of this study was two-fold. First, we examined how CoP groups were defined and used by reviewing primary studies from the two sectors. Second, we assessed the evidence on the effectiveness of CoPs in health care settings.

## Methods

### Search strategy

To identify all existing descriptions of CoP groups in the health and business literature, we used the following strategy to search for studies published between 1991 and 2005:

1. Searching electronic bibliographic databases, including Medline, CINHAL, HealthSTAR, EMBASE, ERIC, ECONLIT, AMED, and ProQuest. The search strings for Medline (Additional file 1) were adapted for other databases.

2. Hand-searching key journals, including *Journal of Continuing Education in the Health Professions*, *Medical Education*, and, *Harvard Business Review*.

3. Examining the reference lists of the included articles and books for additional literature.

In addition, we consulted with members of *CP Square * about the search strategy and the review methodology through two teleconferences on 19 and 23 November 2004. *CP Square *is a 'CoP of CoP' hosted by Wenger and colleagues.

The literature search was conducted in September 2005 by one of the researchers (LL) and a librarian/information scientist (JM). To examined how CoP groups were defined and used, we restricted our search to primary studies involving groups that were either labelled as CoPs or were developed using CoP and/or other related theories (e.g. situated learning, legitimate peripheral learning) as the guiding framework. To examine the evidence on the effectiveness of CoPs in the health sector, we identified articles that: evaluated CoPs for improving health professional performance, health care organizational performance, professional mentoring, and/or patient outcome; and used experimental, quasi-experimental (controlled clinical trials (CCT), interrupted time series (ITS), controlled before-and-after (CBA), or observational designs (before-and-after studies, cross-sectional studies).

The article selection involved a two-phase review. In the first phase, two reviewers (LL and CN) screened the titles and abstracts to identify primary studies that described or evaluated a CoP group. In the second phase, two reviewers, LL and CD (a research coordinator), categorized the included articles into one of five sectors: health care, business, education, information science, and other. All disagreements were discussed, and a third reviewer (MJ) was involved if no consensus was reached.

### Data extraction and analysis

To understand how CoPs were defined and used in the business and health sectors, our literature review was guided by the meta-narrative technique [7,8]. It began by studying the key theoretical publications, reviews, and critique papers; analyzing the key components of a CoP; and using the information to develop a data extraction form. The form was tested on three health sector articles by four research team members (LL, CD, CM, and MJ) and a collaborator from a research funding agency (PM). The content was subsequently modified to capture the interpretation of the CoP concept, and the development, organization, and activities within CoP groups. The final version included the following categories: the study authors' definition of a CoP; duration of the CoP group; members and their disciplinary backgrounds; methods and frequencies of communication; administrative structure; and statements that described 'community,' 'domain,' and 'practice' as defined by Wenger *et al*. [3]. Data extraction of all health sector studies was done independently by two reviewers (LL and CD). The remaining papers were reviewed by CD, and the data were verified by LL.

We conducted concept analysis to explore the interpretation of the CoP concept and the characteristics of CoP groups [9]. The analysis aimed to highlight the similarities and differences in findings across sectors. In this review we focused on three areas: the authors' interpretations of the CoP concept, the key characteristics of CoP groups in primary studies, and the common elements of CoP groups. The characteristics of these groups, reported in primary studies, were summarized in five categories:

1. Why was the group formed?

2. Who was included in the group?

3. How did members communicate?

4. What did the members do or produce, individually or collectively?

5. Where did members interact with each other?

Each sector was reviewed separately, and codes were inductively developed by LL. These codes were uncovered by identifying similarities or differences in phrases, as well as meaningful patterns and processes between and within the different sectors. They were then merged into broader themes. Key reviews and critiques were used to verify the analysis. Throughout the process, the reviewers had frequent discussions and sought input from other team members to identify additional codes and themes. We subsequently discussed the analysis with other researchers with an interest in CoPs for further feedback.

To assess the effectiveness of CoPs, a separate data extraction form was developed to record the following information: number and type of participants, sex, age, the description of CoPs (intervention groups) such as settings and organizational structures, and the description of interventions received by the control group. For each continuous measure, the baseline value and standard deviation were extracted. Also, mean changes from baseline with standard deviations in outcome measures assessed at the end of the treatment period and at the follow-up period were recorded, if available. For dichotomous data, medians and interquartile ranges were recorded at baseline and the subsequent assessments.

Although we were unaware of the number of articles that would meet the eligible criteria, we anticipated extreme heterogeneity among the included studies. Hence, our analysis plan for RCTs, CCTs, and CBAs included calculating standardized effect sizes for the continuous measures, and calculating the number of comparisons showing a positive direction of effect, median effect sizes, and number of comparisons showing statistically significant effects for the dichotomous measures. For the ITS comparisons, the significance of changes in level and slope would be reported. For observational studies, a descriptive summary would be presented.

## Results

**Figure 1:**
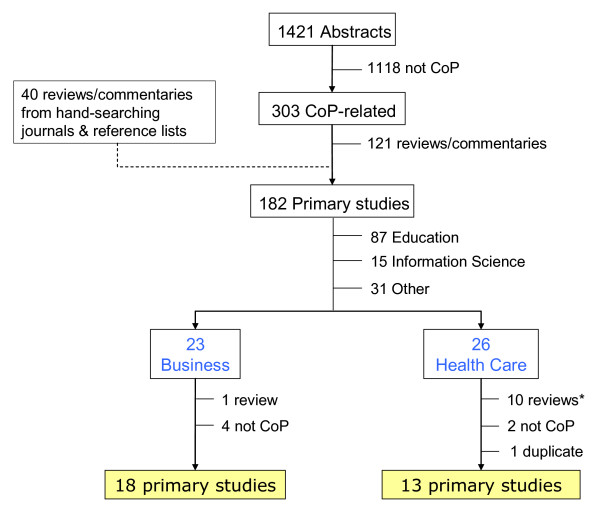
**Literature search strategy**.

**Figure 2:**
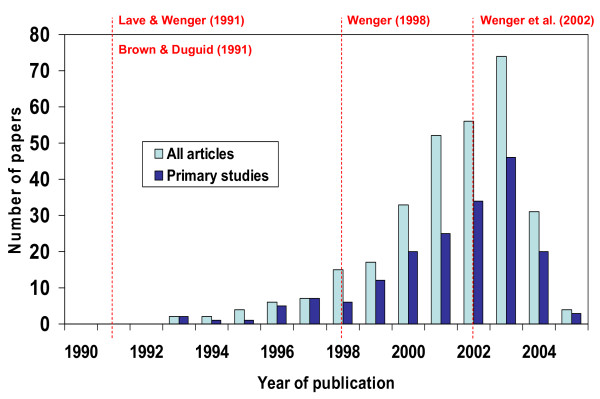
**Number of papers about community of practice (N = 303) and number of primary studies (N = 182) by year**.

The search of electronic databases found 1,421 articles, of which 303 were related to CoPs (Additional file 2; Figure 1). A total of 182 articles were identified as primary studies, and a full review was conducted in 18 primary studies from the business sector [10-27] and 13 from the health sector [5,28-39]. Most of the CoP-related papers were published after 1998, with a publication peak in 2003, after which time the numbers began to decrease (Figure 2). The reason for the decline was unclear, but it should be noted that a few critiques published after 2005 challenged the completeness and usefulness of the CoP for conceptualizing social learning and knowledge management [40-42].

### Communities of practice in business

The term CoP emerged in business literature in the mid-1990s, but articles about social learning and knowledge management had already appeared in journals such as *Harvard Business Review *as early as the early 1990s [43,44]. Most (77.8%) of the primary studies were conducted in the US (Additional file 3). The earlier studies focused on apprentice training, but the term was later used to describe a variety of groups, including formal training sessions [13], informal learning groups [14-16], multidisciplinary teams [17-19], and virtual communities [20-22]. Most studies cited Wenger [1-3], but one [23] referred only to Brown and Duguid [45], and one did not cite any of the seminal work [10].

In 1996, Henning studied refrigeration service technicians and documented the information exchange and mentoring that took place during informal gatherings [14]. In addition to learning and building a professional identity, new workers gained confidence in making work-related decisions. Similar findings were reported by Attwell on the experience of an apprentice in the train re-servicing industry [10]. Harris *et al*. also highlighted the importance of the interaction with mentors in the workplace, which helped new tradespersons make sense of contradictory information that they learned in the classroom [11].

A prominent characteristic of the business CoPs is a willingness to invest time and resources to facilitate activities for members to socialize. While some groups were encouraged explicitly by employers to connect with others on and off the job [14,16,17,20,22], others were provided with communication equipment to enable networking [21,22]. Also, these groups tended to use a range of formal and informal activities. For example, Henning documented the on-the-job meetings and after-work telephone calls among refrigeration technicians [14], Robey illustrated the mix of formal face-to-face meetings and after-work social activities for workers of a soft goods manufacturing company who worked at different sites [22], and Benner described the organized monthly social outing of women working in the information technology companies [16].

### Communities of practice in health care

Most (92.3%) primary studies of health sector CoPs were from the UK or the US. The term 'community of practice' began to surface in this field in the mid-1990s and was often used as a label for groups and teams, rather than a social learning concept. Learning, sharing information, and identity-building were the major focus of these groups, with situated learning and/or legitimate peripheral participation being the guiding concepts.

In 1995, Jenkins and Brotherton published a series of papers on the use of situated learning in the occupational therapy curriculum [46-48]. They argued that occupational therapists consolidate their knowledge and skills most effectively while practising in the clinical setting (i.e. a CoP), and recommended early clinical placements as part of the professional training [46-48]. In a later case study, Lindsay documented the growth of occupational therapy students as they practised applying clinical reasoning skills acquired from a seminar through working with mentors and patients [30]. As students gained experience and confidence in the clinical setting, they were advanced to more complex cases. This process, described by Lave and Wenger as legitimate peripheral participation [1], helped to shape students' career goals and identities as occupational therapists.

In nursing, Cope *et al*. also promoted the use of legitimate peripheral participation as a theory for students to gain skills and professional identity in their clinical placements [5]. The term 'communities of practice' began to appear in the medical literature around 2002 when Parboosing published an opinion article discussing the use of CoP groups to facilitate continuing professional development for physicians [49]. Also, Winkelman and Choo envisioned a CoP as an intervention for patient empowerment [50].

All the primary studies were published in 2000 or later, and the term CoP was used as a synonym for a group of health professionals who are working together. Some authors even argued that a cohesive multidisciplinary team with a clear sense of identity was a CoP [51]. We found that 12 of the 13 primary studies cited Wenger and colleagues' definition of a CoP; however, the actual structure and function of these groups varied greatly. Examples of CoP groups include (Additional file 4): clinical placements where students interacted with and learned from expert practitioners [5,30,52], informal learning groups (e.g. journal clubs [32]), health care agency collaboratives that aimed to achieve a common goal (e.g. to improve primary care for older people [33]), and virtual communities where practitioners from different sites discussed work-related issues [35-38]. Grounded in situated learning and legitimate peripheral participation, studies on clinical placements and apprenticeship tended to focus on students' acquisition of knowledge, skills, and professional identities. However, in groups that focused on information-sharing/-creation, CoP was primarily used as a managerial tool for continuing professional development and improving quality of care, rather than identity development.

Compared to the business sector CoPs, the health care CoPs focus mainly on fostering social interactions at the workplace or during task-oriented activities (e.g. a journal club). Four studies described the use of information technology for members to hold informal discussions and formal meetings [35,36,38,53], but we did not find any study that supported off-the-job social outings.

### Shared characteristics of communities of practice in business and health care

The structures of CoP groups in business and health sectors are summarized in Additional files 5 and 6 respectively. Learning and sharing information through socialization appeared to be the central characteristic of CoP groups. We found all groups demonstrated, to varying degrees, the following characteristics:

1. Social interaction – Interaction of individuals in formal or informal settings, in person or through the use of communication technologies.

2. Knowledge-sharing – The process of sharing information that is relevant to the individuals involved.

3. Knowledge-creation – The processes of developing new ways to perform duties, complete a task, or solve a problem.

4. Identity-building – The process of acquiring a professional identity, or an identity of being an expert in the field.

The knowledge-sharing/-creation CoPs and apprenticeship CoPs emphasized different points, with the latter being focused more on identity-building (e.g. student nurses learning to be a nurse, or new technicians learning to be an expert). Also, it appeared that the mature and cohesive groups tended to include processes that address all four characteristics [14,35,37,54], while the newer groups tended to invest more in activities that encourage social interaction and knowledge-sharing, but less in identity-development or knowledge-creation activities. Also, knowledge-creation was rarely a focus in the apprentice training because the goal was to learn existing skills rather than to develop new ones. While the process of knowledge-sharing could be observed in all CoP groups, the benchmarks for the other three characteristics were less clear in the emergent and maturing CoPs.

### Responsibilities of facilitators

A number of studies from both sectors highlighted the importance of facilitators, and some linked the success and failure of the CoP to this role [15,16,20,24,30,32-36,38,55]. However, the actual responsibilities of facilitators and the organizational support required for this role were less clear in the literature. For example, some facilitators played a distinct role from that of the leader and conducted their activities under the direction of the group and/or the leader [34,35,38], while other groups merged the role of the leader and facilitator [32,55]. The choice of management structure appeared to depend on the size of the group and the availability of human resources. Which model best suited which type of organization was unclear, but facilitator fatigue was mentioned as something that could lead to the downfall of CoP groups [32].

### Power relationships within communities of practice

Ambiguity was observed in the power relationships among CoP members. In the apprenticeship CoPs, the hierarchy of power was usually clearly defined by the roles of mentor-mentee or expert-novice. New practitioners moved from the periphery to a position of full participation as they developed their knowledge and skills by learning from skilled practitioners. Those with full participation would play a greater role, and subsequently had more power to direct the group's activities. In contrast, the power relationship was less clear in the non-apprenticeship CoPs. The inherent assumption was that members of a CoP are naturally collegial, honest, and respectful of each other, and that they put aside their personal agendas for the common good. However, in the non-apprenticeship CoPs, members may not necessarily develop beyond a position of peripheral participation (i.e. they remain as learners/observers rather than contributors), and so learning and negotiation of meaning may continue to be only a reflection of the dominant source of power. This could therefore affect the effectiveness of the group when completing a task or achieving a goal.

One example of people remaining in peripheral participation over the evolution of a CoP group, and therefore of power imbalance, was the multi-stakeholder collaborative in the health sector reported by Gabbay *et al*. [34]. This group was formed to develop health care policies for elder care. Group members participated in scheduled meetings that were organized and facilitated by an experienced librarian. However, despite the facilitator's best efforts, the discussion was often dominated by the opinion and agenda of only a few members. As the group evolved, members like physicians, experienced nurses, and representatives from the health authority were entrusted with more power, and their opinions were valued more by the rest of the group. This subsequently affected the policy development, and some key decisions were based on individuals' experience and preferences rather than the evidence.

### Effectiveness of communities of practice in the health sector

CoP research in the health sector focused mainly on the exploration of how people shared information, created knowledge, and built a professional identity in a social setting. Researchers predominantly used in-depth interviews and participant observations (Additional files 3 and 4). Action research methods, in which participants were involved in the development, growth, and evaluation of the group, were also used [33,34,37]. In this review, we did not find any paper in the health sector that met the eligibility criteria for the quantitative analysis (Additional files 3 and 4); and so the effectiveness of CoP in this sector remained unclear.

## Discussion

The purpose of this study was to describe how CoP groups were defined and used in the business and health sectors, and to assess the evidence on the effectiveness of CoPs in health care settings. One main observation is the wide variety of structures of CoP groups, which range from voluntary informal networks to work-supported formal education sessions, and from apprentice training to multidisciplinary, multi-site project teams. This indicates the broad range of interpretations of the CoP concept within the two sectors. A similar observation was also reported in another recent review of health care CoPs [56]. Our analysis also identified social interaction, knowledge-sharing, knowledge-creation, and identify-building as the common characteristics of CoP groups; although it was unclear how these characteristics were defined in a mature group versus an emergent group.

The majority of studies on CoP groups were qualitative studies that were set out to describe how these groups functioned or to study the complexity of developing and sustaining them (i.e. causal explanation). In contrast, there was a lack of empirical research that examined if CoP groups indeed improved the uptake of best practices in the health sector (i.e. causal description). Perhaps one of the reasons that the CoP has not inspired much evaluative research is that it is actually not a theory of social learning; rather, it is a broad conceptualization of how learning occurs in a social environment, and forms the basis for middle-range theories that are more concrete and address specific problems. However, the process of developing middle-range theories is complicated by the marked divergences in the focus of the CoP concept over the years. The concept originally promotes self-empowerment and professional development [1,2], but as it evolves, it becomes a tool for managing the knowledge flow within organizations with the main purpose of improving organizations' competitiveness [3]. The tension between satisfying individuals' needs for personal growth in the earlier version of the CoP concept versus the organization's bottom line is perhaps the most contentious of the issues that make the CoP concept challenging to interpret and apply [57].

A major limitation of this review was that we only included publications between 1991 and 2005, meaning that there was a four-year lag between the initial literature search and the publication of this paper. Due to the complexity of the data extraction and synthesis, the study took longer than expected to complete. However, because of the significant time gap, it is possible that we have missed important new findings that could inform the field.

Another limitation is that our eligibility criteria only include studies on groups that are labelled as CoPs, and exclude studies that feature teams and groups that do not call themselves CoPs but have the four characteristics. This may be addressed by revising the search criteria and include terms associated with the CoP characteristics; however, because the review was originally designed to assess how the CoP was operationalized in the literature, we chose not to modify the search and review strategy.

Finally, we did not conduct a quality appraisal on the included qualitative studies. The use of quality assessment scales to determine the inclusion of qualitative studies has been a controversial topic. Daly *et al*. have recently proposed a hierarchy of evidence for qualitative studies, with 'generalizable studies' that use a rigorous sampling and analytical approach being the highest level of evidence and single case studies being the lowest [58]. A few tools and frameworks for assessing qualitative studies have also been created [59-61]. However, the reliabilities of these tools within and between reviewers tend to be poor and are no better than simply relying on the unprompted opinions of expert qualitative researchers [62]. The current quality assessment approaches are also criticised as being reductionist and problematic because they often fail to take into account the broader rationale, context, and assumptions of qualitative research [63]. It has also been argued that none of the existing tools are sufficient to incorporate the various conceptions of 'good quality' and 'rightness' [64], and so studies should not be excluded based on the quality assessment. In light of this debate, we decided to include all eligible qualitative studies in this review, regardless of their quality.

This review has identified several areas for further research in order to improve the usefulness of the CoP concept. First, we have identified four common characteristics from CoP groups that were developed over a period of 15 years; the next step will be to develop specific indicators that expand on these characteristics, so that one can distinguish 'CoPs' from 'non-CoPs' and identify the stage of development of a CoP group. Second, there needs to be a better understanding about the expectations, roles, and responsibilities of facilitators and other participants, and the power relationship within CoPs. Wenger and colleagues suggested that an ideal CoP group should include a leader(s)/champion(s), a facilitator(s), a core group of experts who regularly interact with the group, and a dedicated group of members with varying levels of expertise [3]. Future research should explore the specific responsibilities of members in different roles and their interaction in different types of CoPs. Finally, more research will be needed to understand the power relationship within the non-apprenticeship CoPs. This is the subject of a few recent critiques that have pointed out that the lack of clarity on how to handle power dynamics within a CoP has hindered its use as a knowledge-management tool in organizations [42,65].

In conclusion, the CoP remains relevant as a concept to provide guidance for the development of groups, teams, and networks, but it requires further research to develop indicators for identifying CoP groups and for describing the stages of existing and emergent CoPs. We believe that this will enable the development of interventions to facilitate the growth of loosely connected networks to become CoP groups that share and create relevant knowledge, skills, and best practices.

## Competing interests

The authors declare that they have no competing interests.

## Authors' contributions

LL, JG, IG developed the concept. LL, MJ, CN participated in the literature review. LL analysed the data and drafted the manuscript. All authors provided comments and approved the final version.

## Supplementary Material

Additional File 1 **Table S1: Medline search**. The table summarizes the Medline search result. Click here for file

Additional File 2 **Table S2: Electronic database search results**. The table summarizes the literature search results from Medline, CINAHL, ERIC, ECONLIT, AMED, ProQuest, and other sources. Click here for file

Additional File 3 **Table S3: Communities of practice in the business sector – summary of 18 primary studies**. The table summarizes the business sector studies included in the review and their findings. Click here for file

Additional File 4 **Table S4: Communities of practice in the health care sector – summary of 13 primary studies**. The table summarizes the health care sector studies included in the review and their findings. Click here for file

Additional File 5 **Table S5: The structure of community of practice groups in the business sector**. The table summarizes the structure of CoPs in the business sector, in terms of 'why', 'who', 'how', 'what', 'where'. Click here for file

Additional File 6 **Table S6: The structure of community of practice groups in the health care sector**. The table summarizes the structure of CoPs in the health care sector, in terms of 'why', 'who', 'how', 'what', 'where'. Click here for file
