# Supplementary material for: Use of communities of practice in business and health care sectors: A systematic review
Source: Implement Sci. 2009 May 17;4:27. doi: 10.1186/1748-5908-4-27 (PMC2694761; doi:10.1186/1748-5908-4-27)
Supplement: Additional File 1 — Table S1: Medline search. The table summarizes the Medline search result. [file 1748-5908-4-27-S1.doc]

**Table 1: Medline search**

| 1. (communit$ adj practi#e).tw.  2. situated Learning.tw.  3. practice based research network$.tw.  4. primary care research network$.tw.  5. apprenticeship model.tw.  6. legitimate peripheral participation.tw.  7. or/1-6  8. limit 6 to yr = 1991-2005 |
| --- |
