# Supplementary material for: Use of communities of practice in business and health care sectors: A systematic review
Source: Implement Sci. 2009 May 17;4:27. doi: 10.1186/1748-5908-4-27 (PMC2694761; doi:10.1186/1748-5908-4-27)
Supplement: Additional File 2 — Table S2: Electronic database search results. The table summarizes the literature search results from Medline, CINAHL, ERIC, ECONLIT, AMED, ProQuest, and other sources. [file 1748-5908-4-27-S2.doc]

**Table 2: Electronic database search results**

| **Source** | **Retrieved**  **(N = 1421)** | **CoP-related**  **(N = 303)** | **Primary studies on CoPs**  **(N = 182)** |
| --- | --- | --- | --- |
| Medline | 588 | 30 | 19 |
| CINAHL | 202 | 31 | 11 |
| ERIC | 516 | 148 | 77 |
| ECONLIT | 20 | 19 | 3 |
| AMED | 5 | 0 | 0 |
| ProQuest – Dissertation Abstracts | 89 | 75 | 72 |
| Other^†^ | 1 | 0 | 0 |
| ^†^ One article, which was not picked up by the electronic search, was identified by one of the team members during discussions with other CoP researchers. | | | |
