# Supplementary material for: Use of communities of practice in business and health care sectors: A systematic review
Source: Implement Sci. 2009 May 17;4:27. doi: 10.1186/1748-5908-4-27 (PMC2694761; doi:10.1186/1748-5908-4-27)
Supplement: Additional File 3 — Table S3: Communities of practice in the business sector – summary of 18 primary studies. The table summarizes the business sector studies included in the review and their findings. [file 1748-5908-4-27-S3.doc]

**Table 3: Communities of practice in the business sector — summary of 18 primary studies**

| **Type of CoP groups** | **Description** | **Goals** | **Examples from the literature** | **Findings** |
| --- | --- | --- | --- | --- |
| **Apprenticeship** | - Experts interacted with novices in the practice setting. - ‘CoP’ was used synonymously with ‘profession’ or ‘trade.’ | - To learn and consolidate skills. - To acquire a professional identity. | - Attwell (1997): A train re-servicing worker at the Great Western Railway transitioned from an apprentice to a skilled tradesman.[10] (England) - Study design: Case study; face-to-face interview. | - Socialization with other tradesmen was an essential process for a novice to learn skills and gain acceptance by their colleagues. The latter was described as gaining ‘entry into communities of practice.’ |
|  |  |  | - Harris (2003): On-the-job and off-the-job training for new workers in the building and construction industry.[11] (Australia) - Study design: Semi-structured interviews. | - Tradespersons were often required to integrate contradictory knowledge that they learned from the classroom versus on the job. - CoPs appeared to provide a structure whereby new workers could make sense of contradictory information by working with more experienced workers. |
|  |  |  | - Machles (2004): Employees of a biotechnology company learning about occupational safety practices.[12] (US) - Study design: Semi-structured interviews. | - Participants felt that learning occupational safety practices occurred through experience with equipment and within their work environment. - Workers learned from their mentors and peers by sharing stories about occupational safety practices. |
| **Formal education program** | - Professionals interacted with instructors and peers in an adult education program. | - To acquire work-related knowledge and the identity of an ‘expert.’ | - Ball (2003): Trade union representatives participated in education programs to improve their knowledge and ability to represent their fellow members. The program was attended by both experienced and new members.[13] (UK) - Study design: Mail survey, interviews, life-history narratives. | - Formal and social interaction with experienced members helped newcomers to gain knowledge, skill, and confidence to act as a union representative. |
| **Informal network** | - Professionals interacted in informal, social settings to share trade-related information. - Most meetings were face-to-face. | - To share information so that people can do their own job better. | - Henning (1996): Seven refrigerating technicians, at various level of expertise, met socially to share information about work.[14] (US) - Study design: Ethnographic field study. | - Social interactions among these technicians enabled them to improve skills, and helped the newcomers to develop a professional identity. |
|  |  |  | - Yi (2000): Engineers at Motorola participated in an informal community called SMART CoP.[15] (US) - Study design: Case study. | - Engineers noted that being a member of a CoP was beneficial for their learning and professional development. - Participation in the CoP increased their awareness of the need for collaboration and sharing. - Clear ground rules, roles, and responsibilities were important for members to establish boundaries and for orienting newcomers. |
|  |  |  | - Benner (2003): Women in Internet design and development occupations participated in a group called Webgrrl. Members interacted through listserv and monthly social outings to share tips about working in the industry.[16] (US) - Study design: Case study. | - The informal interactions enabled members to share information, to exchange job and business leads, and to learn about new technologies. This was particularly valuable to newcomers. - The high levels of trust and openness contributed to the success of this group. |
| **Multidisciplinary team** | - People from different disciplines, who normally would not work together, collaborated on a common task. | - To develop/build a product. | - Schermer (2002): Chrysler commissioned a group of professionals to design and build the Chrysler Technology Centre. Architects, designers, and construction professionals met formally and socially to discuss work-related issues and problems.[17] (US) - Study design: Case study; face-to-face interviews. | - Architects in a commissioned project had to work with professionals from other disciplines and organizations. CoP-type groups helped them to learn about each other’s expertise, viewpoints, and working style, which helped to facilitate the progression of the project. |
|  |  |  | - Carlson (2003): A proposal-writing team, consisting of scientists, engineers, technical managers, editors, graphic artists, text processors, and production coordinators, collaborated in a research and applied engineering laboratory.[18] (US) - Study design: Case study; face-to-face interviews. | - The research discovered five processes that occurred in a proposal-writing CoP: owning, visioning, producing, contributing, and reviewing, all of which are vital to the maturation of the CoP. - Newcomers benefited from the mentoring provided by the experienced team members. |
|  |  |  | - Barrett (2004): Engineers and workers from different divisions of a shop that produced moulds for packaging and bicycle helmets engaged in learning by participating in informal groups.[19] (US) - Study design: Semi-structured interviews. | - A CoP-type team treated the participation in a social setting as a part of learning and identity building for new workers. - Class distinction among workers could add stress and competition within a workplace, which in turn hindered social participation and learning. |
| **Virtual community** | - Online groups for practitioners to discuss issues related to work or the trade. | - To help participants from various geographic locations to do their work better. | - Ardichvili (2002): A Fortune 100 construction and mining equipment company developed >600 online groups for 16,000 employees worldwide to facilitate knowledge-sharing.[20] (US) - Study design: Case study; face-to-face interviews. | - Elements contributing to the success of an online CoP included: the corporate culture of knowledge-sharing, the belief that knowledge belongs to the whole organization, freedom for employees to organize new CoPs around specific performance-related problems or professional interests, and the presence of competent CoP facilitators and the active participation of content experts.   Barriers included: discomfort about posting something online for all to see, security issues, concerns about the accuracy of the information, and potential information overload. |
|  |  |  | - Beamish (2000): Ford developed an online distance education system, Fordstar, for salespersons, mechanics, and parts and service personnel within dealerships.[21] (US) - Study design: Participant observation, documents analysis, interviews. | - The availability of online technology alone did not ensure information flow to the end users. - Obstacles to information flow could include: content (positive vs. threatening), medium (ease of use), physical environment (noise, transmission quality), cultural and social environment, work environment (salary system, competition), and individual issues (position, beliefs, attitudes, memory, ability to act). |
|  | - Teams, often from the same company, collaborated from different locations by using communication technologies. | - To share information and complete assignments. | - Robey (2000): Members of three cross-functional virtual teams from a soft goods manufacturing company worked and learned together through e-mails, phone calls, fax, videoconferencing, and face-to-face meetings [22]. (US) - Study design: Face-to-face, semi-structured interviews. | - A virtual community can facilitate learning among workers from different geographic locations. - Online interactions reduced the need for face-to-face meetings, while maintaining the level of productivity and learning. |
| **Primary studies that used CoPs as a learning theory** | - The term ‘CoP’ was used in the study, but there was no information about the structure of the group or the effect on participants. | 1. Gieskes (2002): The learning style and barriers to learning in a multi-national telecommunication company were examined. CoP was discussed as a learning theory.[23] (Multinational: the Netherlands, Sweden, Australia) 2. Beers (2003): The role of knowledge-brokering was examined in a qualitative study. CoP was used as the underlying theory of social learning. It was proposed that knowledge brokers might help to increase collaborations among CoP-type groups. [24] (US) 3. Boyer (2003): A survey was administered to the employees of a computer service business to assess the extent to which the CoP theory supported the company’s competence in knowledge-management. [25] (US) 4. Kull (2003): A case study that compared CoP theory and other knowledge-management concepts. Twenty-eight knowledge-management experts were interviewed.[26] (US) 5. Sinha (2004): A Web-based survey was administered to managers and workers of 42 organizations, including banks, engineering firms, information technology companies, and manufacturing companies, to assess whether the existence of CoP-type groups increase an organization’s competitive advantage.[27] (US) | | |
| **Legend:**  CoP = Community of practice | | | | |
