# Supplementary material for: Use of communities of practice in business and health care sectors: A systematic review
Source: Implement Sci. 2009 May 17;4:27. doi: 10.1186/1748-5908-4-27 (PMC2694761; doi:10.1186/1748-5908-4-27)
Supplement: Additional File 4 — Table S4: Communities of practice in the health care sector – summary of 13 primary studies. The table summarizes the health care sector studies included in the review and their findings. [file 1748-5908-4-27-S4.doc]

**Table 4: Communities of practice in the health care sector — summary of 13 primary studies**

| **Type of CoP groups** | **Description** | **Goals** | **Examples from the literature** | **Findings** |
| --- | --- | --- | --- | --- |
| **Apprenticeship** | - Expert practitioners interacted with novices in the practice setting. - ‘CoP’ was used synonymously with ‘profession.’ | - To learn and consolidate clinical skills. - To acquire a professional identity. | - Cope (2000),[5] Burkitt (2001)[28]: Student nurses learned from mentors and other nurses during clinical placements (UK, US). - Study design: Semi-structured interviews, focus groups, field observations. | - Students gained acceptance in the workplace through interacting with mentors and colleagues. |
|  |  |  | - Hudzicki (2004): The transition of medical technologists from novices to experts.[29] (US) - Study design: Semi-structured interviews. | - Transition from novice to expert required individuals to be self-directed and reflective, and to have access to mentors. The latter required being a member of a CoP. |
|  |  |  | - Lindsay (2000): Fieldwork program at a community mental health centre for junior OT students.[30] (US) - Study design: Case study. | - Through participation in individual meetings with clinical instructors and team meetings, students reported an increase in confidence in clinical reasoning and in setting career goals and objectives. |
|  |  |  | - Plack (2003): PT students and novice clinicians transitioned from an academic to clinical setting.[31] (US) - Study design: Semi-structured interviews and questionnaires, focus groups. | - Active engagement in practice and dialogue with experienced clinicians was critical for novices to form their professional values, beliefs, attitudes, and identities. |
| **Informal learning group** | - Groups of clinicians engaged in continuing professional development activities. | - To share knowledge so that members can do their own job better. | - Pereles (2002): Physicians met regularly at journal clubs.[32] (Canada) - Study design: Semi-structured interviews. | - Members of these groups appeared to be supportive of each other’s learning. - Mutual respect was a major contributor to the success of a group. Members preferred to agree to disagree rather than pursue a ‘right’ answer or consensus. - Facilitators in these groups played a key role in providing administrative support. Burnout was an issue that could lead to the demise of these groups. |
| **Health care agency collaborative** | - Representatives of health care agencies, who normally would not work together, collaborate to achieve a common task. | - To provide quality health care. | - Lathlean (2002),[33] Gabbay (2003)[34]: Multi-agency groups with a mandate to develop evidence-based policies to improve health and social services for older people. (UK) - Study design: Action research. | - Even with challenges, CoPs might improve interagency communication and local services.[33] - Policy was not always developed based on the research evidence, even within a well-facilitated CoP. Decisions might be heavily influenced by the power dynamics within the group.[34] |
| **Virtual community** | - Online groups that helped practitioners to use/adopt an innovation (*e.g.*, a new tool, new guidelines), or to discuss practice-related issues. | - To help participants from various geographic locations to do their work better. | - Wild (2004): Eleven states and local public health agencies, which engaged in developing and implementing a children’s health information system, participated in Connections, an online forum, to share best practices.[35] (US) - Study design: Web-based survey, individual and group interviews. | - Members felt that *Connections* offered a safe haven where they felt comfortable sharing their successes and failures without the fear of being judged. - Membership diversity was considered a strength. - Site visits were the most useful activity for both the hosts and the visitors. - Technical challenges hindered the use of the interactive website by members. |
|  |  |  | - Richardson (2003): A Web-based network, Health Voice, for students of post-graduate degree programs in health disciplines to facilitate inter-professional collaboration.[36] (UK) - Study design: Program evaluation, interviews. | - *Health Voice* provided an alternative medium to face-to-face interaction for learning and for developing an identity as a member of an inter-professional learning group. |
|  |  |  | - Tolson (2005): A ‘virtual practice development college’ for gerontological nurses across the country. Thirty-six nurses and academics interacted in this Web-based group and at real-time meetings for two years.[37] (UK) - The group developed, piloted, published, and implemented evidenced-based nursing care recommendations. - Patients and their families were involved where possible. - Study design: Action research. | - Nurses felt that being a member of a national CoP afforded status and strengthened their sense of professional identity. - Discussions with other nurses helped participants to approach best practices from the nursing perspective, in addition to the traditional medical perspective. - Major challenges of being in a virtual CoP included the absence of a ‘learning-at-work culture,’ lack of time, and doubts about the legitimacy of Internet-based learning. |
|  |  |  | - Russell (2004): CHAIN, an informal e-mail network for people working in health care, or with an interest in evidence-based health care, to share expertise, make new contacts, and provide mutual support.[38] (UK) - Study design: Illuminative evaluation. | - The network served as a rich source of information by providing access to members’ experiences, suggestions, and ideas. - Ad hoc groupings emerged spontaneously as members discovered common areas of interest. - A skilled facilitator and support staff served an important role in linking members with the same interests. |
|  |  |  | | |
| **Primary studies that used CoP as a learning theory** | - The term ‘CoP’ was used in the study, but there was no information about the structure of the group or the effect on participants. | | - Haigh (2004): This study described a ‘community of communities’ as a peer evaluation process to maintain service standards in community mental health organizations. [39] [UK] | |
| **Legend:**  CoP = Community of practice  OT = Occupational therapy  PT = Physical therapy  CHAIN = Contact, Help, Advice and Information Network for Effective Health Care | | | | |
