# Supplementary material for: Use of communities of practice in business and health care sectors: A systematic review
Source: Implement Sci. 2009 May 17;4:27. doi: 10.1186/1748-5908-4-27 (PMC2694761; doi:10.1186/1748-5908-4-27)
Supplement: Additional File 5 — Table S5: The structure of community of practice groups in the business sector. The table summarizes the structure of CoPs in the business sector, in terms of 'why', 'who', 'how', 'what', 'where'. [file 1748-5908-4-27-S5.doc]

**Table 5: The structure of community of practice groups in the business sector**

| **Reference** | **Why was the group formed?** | **Who was included in the group?** | **How did members communicate?**  **What did the members do or produce, individually or collectively?** | **Where did members interact with each other?** |
| --- | --- | --- | --- | --- |
| **Henning (1996)[14]** | To become a refrigeration technician; to develop new ways to solve technical problems. | Novice and expert refrigeration technicians. | **How:** Roles of mentors and mentees were pre-determined.  **What:** Students learned the use of trade jargons by interacting with expert technicians. | Formal one-on-one meetings; off-work telephone calls. |
| **Attwell (1997)[10]** | To become a skilled train re-servicing worker. | An apprentice learning to refurbish railway cars, and the foreman. | **How:** Roles of mentors and mentees were pre-determined.  **What:** The apprentice acquired the complex skill of refurbishing railway cars. | The mentees work alongside the mentors every day at the shop. |
| **Robey (2000)[22]** | To share knowledge. | Office workers from two geographic locations. | **How:** Individuals traveled to business meetings, and then met socially after the meetings.  **What:** Workers supported each other to reach their performance goals. | Videoconference, formal and informal meetings, telephone calls. |
| **Yi (2000)[15]** | To complete a task: to build an online learning tool. | Engineers at Motorola. | **How:** The group started with a kick-off meeting, followed by facilitated online chats. Members collaborated on specific projects and presentations.  **What:** The group archived online questions and responses. | Online discussion groups through the intranet. |
| **Ardichvili (2002)[20]** | To share knowledge and solve work-related problems. | Engineers at the Caterpillar company. | **How:** Employees posted questions on the intranet. Community managers forwarded the questions to experts in various disciplines and initiated discussions.  **What:** Employees collaborated on projects and discussed work-related issues. | E-mails and intranet online forums. |
| **Beamish (2002)[21]** | To share knowledge | Car salespersons and service technicians at a Ford dealership. | **How:** Peer learning on increasingly difficult tasks; Ford sent out newsflashes on problems and solutions reported by dealerships.  **What:** Documents on best practices were shared. | Satellite broadcasts,  face-to-face meetings. |
| **Schermer (2002)[17]** | To complete a task: to build a building for Chrysler. | Architects, engineers, construction managers, clients (representatives from Chrysler) | **How:** Team member interaction was facilitated by formal training sessions on teamwork, the code of conduct, and the design of the office (*e.g.*, low partitions in the office space to encourage conversations).  **What:** Members exchanged work-related information. | Formal group meetings, informal discussions at worksite. |
| **Ball (2003)[13]** | To become a competent trade union representative. | New and experienced trade union representatives. | **How:** Roles of mentors and mentees were self-selected.  **What:** New union representatives learned from the experienced ones about the role. | Weekly trade union education session, informal discussions with peers. |
| **Benner (2003)[16]** | To share knowledge. | Women working in Web-related occupations in Silicon Valley. | **How:** Old members greeted new members at social events and oriented them toward the online activities; members also get together informally every month.  **What:** Members shared technical, career, and business advice. | E-mails, listserv, scheduled social events. |
| **Carlson (2003)[18]** | To complete a task: to produce a proposal. | Scientists, engineers, graphic artists. | **How:** Team members collaborate to produce a proposal. They provide constructive critiques for each other.  **What**: Meeting minutes were kept as both electronic and hard copies. | Formal group meetings. |
| **Harris (2003)[11]** | To become a competent tradesperson. | New workers and instructors in the building and construction industry. | **How:** Roles of mentors and mentees were pre-determined.  **What:** Through classroom and on-the-job interactions with the instructors, new workers gained independence and confidence in their skills and roles. | Small group meetings,  on-the-job learning from experienced workers. |
| **Barrett (2004)[19]** | To produce a good product. | Engineers and labourers at a shop that produced moulds for packaging and bicycle helmets. | **How:** Members from different disciplines collaborated at the shop to produce a product.  **What:** A collection of work-related stories, training modules, and databases that can be used by the current and new employees. | Informal daily interaction at work. |
| **Machles (2004)[12]** | To become competent in work safety practices. | Employees at a biotechnology company. | **How:** Employees learned about occupational safety practices. Role modeling – roles of mentors and mentees were self-selected.  **What:** A collection of occupational safety-related stories from workers, friends, and acquaintances. | Small group sessions, informal discussions with peers. |
