# Supplementary material for: Use of communities of practice in business and health care sectors: A systematic review
Source: Implement Sci. 2009 May 17;4:27. doi: 10.1186/1748-5908-4-27 (PMC2694761; doi:10.1186/1748-5908-4-27)
Supplement: Additional File 6 — Table S6: The structure of community of practice groups in the health care sector. The table summarizes the structure of CoPs in the health care sector, in terms of 'why', 'who', 'how', 'what', 'where'. [file 1748-5908-4-27-S6.doc]

**Table 6: The structure of community of practice groups in health care sector**

| **Reference** | **Why was the group formed?** | **Who was included in the group?** | **How did members communicate?**  **What did the members do or produce, individually or collectively?** | **Where did members interact with each other?** |
| --- | --- | --- | --- | --- |
| **Cope (2000)[5]** | To become nurses | Clinical nurse instructors and nursing students. | **How:** Roles of mentors and mentees were pre-determined.  **What:** Students learned by doing, observing, and discussing with mentors. | One-on-one meetings. |
| **Lindsay (2000)[30]** | To become occupational therapists | Clinical instructors and occupational therapy students. | **How:** Roles of mentors and mentees were pre-determined.  **What:** Students learned by doing, observing, and questioning the mentors. | Scheduled one-on-one meetings, small group discussion. |
| **Lathlean (2002)[33]**  **Gabbay**  **(2003)[34]** | To complete a task:  Create policies | Government agencies, hospitals, social services, voluntary sector organizations, local citizens. | **How:** Members met at formal meetings to discuss a list of topics pre-determined by the group. Communications among members were guided by a facilitator.  **What:** Members exchanged stories based on their work-related experiences and developed a framework for shared decision-making. | Scheduled group meetings. |
| **Pereles (2002)[32]** | To share knowledge | Physicians. | **How:** Small group learning facilitated by a coordinator who provides group material.  **What:** Members shared stories based on practice experience. | Scheduled small group meetings. |
| **Plack (2003)[31]** | To become physical therapists | Clinical instructors and supervisors, physical therapy students, and new physical therapists. | **How:** Roles of mentors and mentees were pre-determined.  **What:** Students learned from instructors by doing, observing, and sharing stories. | One-on-one meetings. |
| **Richardson (2003)[36]** | To share knowledge, and to develop an identity as a member of an inter-disciplinary learning group. | Post-graduate students in a health discipline. | **How:** Facilitator plans and organizes meetings. Students also planned their own online and off-site meetings with other students and faculty members.  **What:** Attend online seminars; students gained feedback from researchers. | Online seminars,  off-site group meetings. |
| **Tolson (2003)[37]** | To share and create knowledge. | Gerontology nurses. | **How:** Problem-solving as a group.  **What:** Members developed, piloted, and published practice statements. | An online college. |
| **Russell (2004)[38]** | To share knowledge. | Researchers and practitioners working in health care-related areas. | **How:** CHAIN staff forward e-mails to appropriate group members. Members may also contact or consult with each other directly, or by asking CHAIN staff to initiate an introduction.  **What:** Facilitator pre-screened all incoming e-mails and forwarded to the relevant members. Members share best practices and network with each other. | E-mail network. |
| **Wild (2004)[35]** | To complete a task: Integration of child health services, finding new ways to solve problems, sharing and creating new knowledge. | Local, State, and Federal Child Health Agencies. | **How:** Member agencies engaged in planning and developing projects. They reviewed each other’s work and collaborated on projects.  **What:** Produced newsletter and presentation CDs. | Teleconferences, e-mails, listserv, group meetings, planned social events. |
